# The tiered-evaluation of the effects of transgenic *cry1c* rice on *Cyrtorhinus lividipennis*, a main predator of *Nilaparvata lugens*

**DOI:** 10.1038/srep42572

**Published:** 2017-02-16

**Authors:** Yu Han, Fugang Ma, Muhammad Nawaz, Yu Wang, Wanlun Cai, Jing Zhao, Yueping He, Hongxia Hua, Yulan Zou

**Affiliations:** 1 Hubei Insect Resources Utilization and Sustainable Pest Management Key Laboratory, College of Plant Science and Technology, Huazhong Agricultural University, Wuhan, China; 2 College of Life Science and Technology, Huazhong Agricultural University, Wuhan, China

## Abstract

T1C-19, a newly developed transgenic *cry1C* rice line, expresses *cry1C* under the control of the maize ubiquitin promoter, and is highly resistant to lepidopteran pests of rice. *Cyrtorhinus lividipennis* is the major predator of the eggs and young nymphs of *Nilaparvata lugens*, which is the main non-target sap-sucking insect pest of *Bt* rice. *C. lividipennis* may be exposed to Cry1C protein, thus biosafety evaluations of transgenic *cry1C* rice on *C. lividipennis* should be conducted before the commercialization of T1C-19. In the current study, we tested the direct toxicity of elevated doses of Cry1C to *C. lividipennis*, effects of T1C-19 on the life-table parameters of *C. lividipennis* via preying planthoppers, and effects of T1C-19 on the population density and dynamics in rice fields. No detrimental effects on development, survival, female ratio and body weight of *C. lividipennis* were caused by direct exposure to elevated doses of the Cry1C protein or prey-mediated exposure to realistic doses of the protein. The population density and dynamics did not significantly differ between *C. lividipennis* in T1C-19 and non-transgenic rice fields. Thus, transgenic *cry1C* rice had no negative effects on *C. lividipennis*. This is the first report of the effects of transgenic *cry1C* rice on *C. lividipennis*.

Rice, *Oryza sativa* L., is an important cereal crop cultivated worldwide. China is one of the largest rice producing countries in the world and it is estimated that the total area under rice cultivation is ~29.4 million hectares, which covers one-third of the total food crop growing area of China. The total rice production of China is ~207.440 million tons, the highest worldwide^1^. China must increase its rice yield to 7.85 × 10^3^ kg.ha^−1^ by 2030 to fulfill its own country’s requirements^2^.

Insect pest control is the greatest challenge to increasing rice yields. In Asian rice-ecosystems, many insect pests, such as the rice planthoppers *Nilaparvata lugens* (Stål), *Sogatella furcifera* (Horváth), and *Laodelphax striatellus* (Fallén) (all Hemiptera: Delphacidae), stem borers *Chilo suppressalis* (Walker) and *Scirpophaga incertulas* (Walker) and the leafroller *Cnaphalocrocis medinalis* (Guenée) (all Lepidoptera: Pyralidae) can cause a tremendous economic losses. Although many other control strategies, such as good farming practices and biological control, have been developed to reduce insect pest-associated economic losses in China, synthetic pesticide spraying is still the main method to control those insect pests. The use of large amounts, as well as the long-term applications, of these chemicals has resulted in environmental contamination and resistance to the pesticides^3^,^4^. Therefore, alternative environmentally friendly and economical pest control-strategies need to be developed.

Genetically modified rice varieties, which express the insecticidal δ-endotoxin (Cry protein) derived from the bacterium *Bacillus thuringiensis (Bt*), exhibit high resistances against rice lepidopteran insect pests. *Bt* rice has been considered as the best alternative to chemical insecticides against rice lepidopteran insect pests in China^5^,^6^,^7^,^8^,^9^. *Bt* rice results a 6–9% increase in yield and 80% reduction in insecticide use as compared with conventional varieties in China^10^.

A series of rice lines expressing various *Bt* genes (such as *cry1Ab, cry1Ab/1Ac, cry1C* and *cry2A*) have been developed to suppress the infestations of target lepidopteran insect pests in China^11^,^12^,^13^,^14^. However, concerns regarding their potential impacts on non-target herbivores and their natural enemies through tritrophic interactions have been raised. Thus, it is necessary to conduct environmental risk assessments prior to their commercial cultivation, and the environmental risks of each rice line must be evaluated on a case-by-case basis and tiered-tests should be conducted^15^. These tests are initiated to evaluate the direct toxicity of elevated doses of insecticidal compounds (e.g., ten times higher than the realistic exposure) to the non-target arthropod (NTA). In this Tier-1 test, purified Bt protein is delivered to the NTA by being mixed with the artificial diets; then in a semi-field test (effects of insecticidal proteins at realistic doses through the food-chain on the NTA in a replicated controlled system); and the field test (effects of transgenic crops on population of NTAs at realistic doses in a realistic agricultural system)^15^,^16^,^17^.

The rice planthoppers are a key group of non-target sap-sucking insects on *Bt* rice that presents lepidoptera-resistance^18^. *Cyrtorhinus lividipennis* (Hemiptera: Miridae) is a major predator of the eggs and nymphs of planthoppers, which regulates the population density of planthoppers in rice fields^4^,^19^,^20^. Based on its ecological importance, the effects of transgenic *Bt* rice on *C. lividipennis* should be determined before the commercialization of *Bt* rice. T1C-19 is a newly developed transgenic *cry1C* rice line that exhibits high resistance against lepidopteran insect pests on rice^14^. Up to date, there are no reports on the effects of transgenic *cry1C* rice on *C. lividipennis*. In the current study, we tested the direct toxicity of elevated doses of Cry1C protein to *C. lividipennis*, the effects of transgenic *cry1C* rice on the life-table parameters of *C. lividipennis* via preying planthoppers, and the effects of T1C-19 on population density and dynamics in rice fields. We also examined Cry1C transduction from rice plants to *N. lugens* and *C. lividipennis*.

## Results

### Fitness of *C. lividipennis* feeding on the artificial diet of *C. lividipennis*

An artificial diet of *N. lugens*, Diet-1^21^, was modified by the addition of eggs of *Corcyra cephalonica* for *C. lividipennis*. This modified artificial diet, Diet-2, was prepared for the Tier-1 test. After feeding natural food and Diet-2, 90.6% and 81.3% *C. lividipennis* nymphs survived and developed to adults, respectively (Table 1). Although the developmental duration of larvae fed with Diet-2 was significantly longer than those fed with natural food, no significant difference was found in the female and male weights between the two diets (Table 1). Thus, Diet-2 could maintain the normal survival and development of *C. lividipennis*. Compared with Diet-1, Diet-2 was more beneficial to the life-table parameters of *C. lividipennis*. The nymphal survival rate was higher, the preimaginal development was faster, and the body weights of adults were greater than those reared with Diet-1 (Table 1). Diet-2 was used for the next Tier-1 test.

### Bioassay with *Galanthus nivalis* agglutinin (GNA)

**Figure 1:**
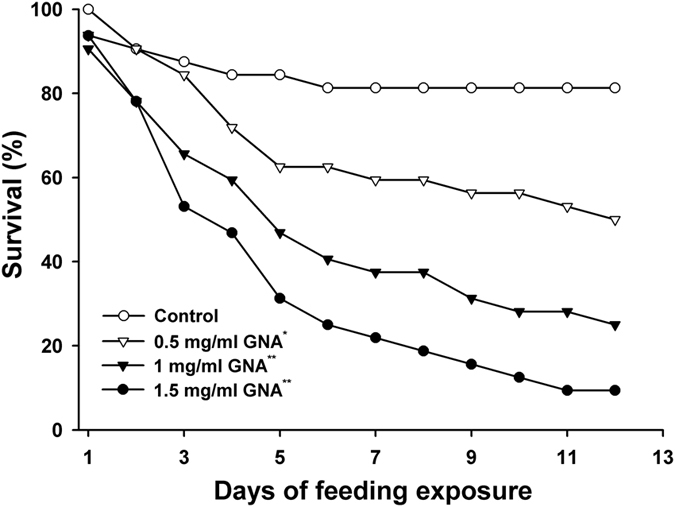
Survival of *C. livdipennis* fed pure Diet-2 or Diet-2 containing different concentrations of GNA (0.5 mg/ml, 1 mg/ml and 1.5 mg/ml). Pure Diet-2 served as a negative control (n = 32). Asterisk denotes a significant difference between GNA treatment and the negative control: ^*^P < 0.05, ^**^P < 0.01, n = 32.

To validate the appropriateness of the artificial diet used in a dietary test system for assessing the toxicity of insecticidal compounds to *C. lividipennis*, GNA, a lectin isolated from snowdrop bulbs, was selected as a model compound because preliminary experiments in our laboratory indicated that GNA is toxic to *C. lividipennis. C. lividipennis*’ nymphal survival rates steadily decreased when fed a diet mixed with increasing GNA concentrations (Fig. 1). A survival analysis indicated that the nymphs fed on any diet containing GNA had significantly reduced survival rates compared with those fed on the control (pure artificial food) (*P *< 0.01; Fig. 1). Under the 0.5, 1 and 1.5 mg/ml GNA treatments, only 46.9%, 21.9% and 9.4%, respectively, of the nymphs reached the adult stages, and the survival rates of these nymphs were significantly lower (*P* < 0.01) than that of the control (Table 2). The duration of nymphal development was also significantly delayed with the incorporation of different GNA concentration in the pure diet. However, there were no negative effects of GNA on the body weights of adults if the nymphs survived to adulthood (Table 2).

### Bioassay with Cry protein

**Figure 2:**
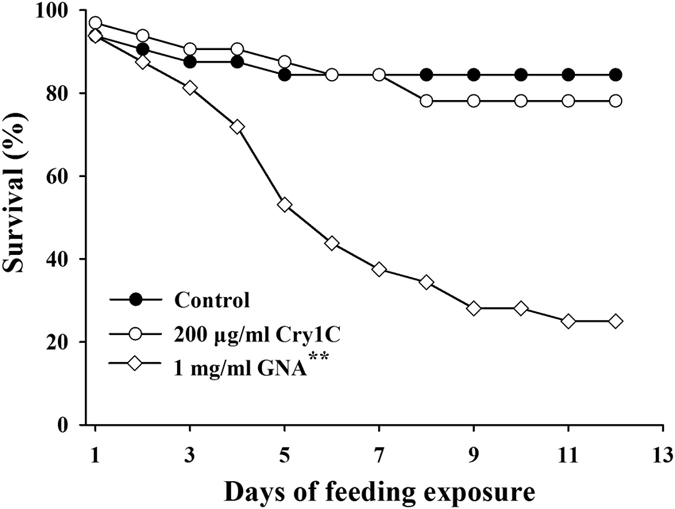
Survival of *C. lividipennis* fed pure Diet-2 or Diet-2 containing Cry1C (200 μg/ml) or GNA (1 mg/ml). GNA was served as positive control. Pure Diet-2 served as a negative control (n = 32). Asterisk denotes a significant difference between a treatment and the negative control: ^**^P < 0.01.

A dietary exposure assay was used to evaluate the direct toxicity of high dosages of Cry proteins to *C. lividipennis*. Second-instar nymphs of *C. lividipennis* were individually fed an artificial diet that contained 200 μg/ml of Cry1C protein. More than 78% of *C. lividipennis* nymphs reached the adult stage when fed on artificial diets incorporating the Cry1C protein (Table 3). In contrast, only 25% of the nymphs developed into adults in the 1 mg/ml GNA treatment, which was significantly less than the control (*χ*^2^ = 22.763, *df* = 1, *P* < 0.001; Table 3). The survival analysis showed that there was no significant difference between the Cry1C treatment and the pure diet (control) (*χ*^2^ = 0.301, *df* = 1, *P* = 0.584; Fig. 2). A significantly lower survival rate was found for the insects feeding on an artificial diet containing GNA compared with those fed the control diet (*χ*^2^ = 18.858, *df* = 1, *P* < 0.001; Fig. 2). Similarly, no differences were detected in the developmental duration (Mann–Whitney *U*-test, *U* = 327.5, *P* = 0.846) and adult fresh weight between the Cry1C and control treatment, while the developmental duration (Mann–Whitney *U*-test, *U* = 21.0, *P* < 0.001) was significantly prolonged, and the female fresh weight was significantly decreased by the GNA treatment in comparison with the pure diet treatment (Table 3).

### Prey-mediated effects of transgenic *cry1C* rice on the life-table parameters of *C. lividipennis*

The life-table parameters, including developmental time, preimaginal survival, female ratio and fresh body weight of adults, did not differ between *C. lividipennis* reared with eggs or nymphs of *N. lugens* fed on T1C-19 and those eggs or nymphs of *N. lugens* fed on Mighui 63 (*P* > 0.05; Tables 4 and 5). The contents of Cry1C in rice, eggs of *N. lugens*, nymphs of *N. lugens* and *C. lividipennis* are shown in Table 6. The Cry1C contents in T1C-19 sheaths and *N. lugens* nymphs were 1.8 ± 0.1 μg/g and 0.6 ± 0.05 ng/g, respectively (Table 6). Cry1C could be transferred to the *N. lugens* nymphs, but the Cry1C content in *N. lugens* nymphs decreased dramatically compared with that of T1C-19 (Table 6). No Cry1C was detected in the eggs of *N. lugens*. If T1C-19 and *N. lugens* nymphs were supplied to *C. lividipennis* together, then Cry1C could be transmitted to *C. lividipennis*. However, if *N. lugens* nymphs were supplied to *C. lividipennis* without T1C-1, then Cry1C could not be transferred to *C. lividipennis* through the *N. lugens* nymphs. Cry1C could be transferred to *C. lividipennis* when eggs of *N. lugens* and T1C-1 were provided to *C. lividipennis* simultaneously, while no Cry1C was detected in *C. lividipennis* when the eggs of *N. lugens* alone were provided to *C. lividipennis* (Table 6). As was expected, no Cry1C protein was detected in the Minghui 63 rice plants (Table 6).

### Effects of transgenic *cry1C* rice on the population density and dynamics of *C. lividipennis*

**Figure 3:**
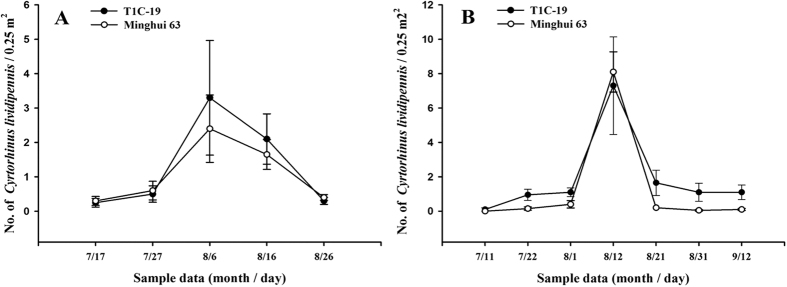
Population dynamics of *C. lividipennis* collected by vacuum-suction. Data are represented as mean ± SE. (**A**) Xiaogan, 2012; (**B**) Xiaogan, 2013. There was no significant difference between *Bt* rice and control plots at the same sampling time, based on Student’s *t*-test (N = 4). Repeated measures ANOVA: (**A**) T1C-19 vs Minghui 63: F_1,6_ = 0.557, *P* = 0.484. (**B**) T1C-19 vs Minghui 63: F_1,6_ = 1.027, *P* = 0.350.

The population densities of *C. lividipennis* in T1C-19 rice fields did not differ significantly in comparison with those in Minghui 63 rice fields, at any site in any year (Student’s *t*-test, *P* > 0.05; Table 7). Similarly, there were no significant differences in population dynamics between Minghui 63 and transgenic *cry1C* rice fields at any sampling date, at any site in any year (Student’s *t*-test, *P* > 0.05; Fig. 3). Repeated measures ANOVA analysis showed that the population dynamics were unaffected by rice line (*P* > 0.05).

## Discussion

*N. lugens* is the main non-target sap-sucking insect pest of transgenic *Bt* rice, and *C. lividipennis* is a major predator of the eggs and young nymphs of *N. lugens*. The potential effects of transgenic *Bt* rice on *C. lividipennis* should be evaluated before the commercialization of any novel *Bt* rice. In the tiered-tests of ecological risk assessment on an insect-resistant transgenic crop for an NTA, “Tier-1 assays” are the initial step to determine the direct toxicity of the insecticidal compounds expressed by the transgenic crop on NTAs. In the present study, we constructed a Tier-1 system to detect the potential effects of high doses of Cry1C on *C. lividipennis*.

In Tier-1 assays, artificial diets are important factors and should meet the following requirements: (i) capable of sustaining normal survival and development of the test species; (ii) test compounds can be readily and uniformly incorporated into the diet; and (iii) test compounds should be active during the feeding exposure duration^16^,^17^. According to a previous report, the artificial diet for rearing *N. lugens* could sustain the survival and development of *C. lividipennis*^22^, but the preimaginal survival of *C. lividipennis* nymphs was only 55%, which needed improvement to meet the survival requirements for a Tier-1 assay (>80% survival)^17^. In the current study, eggs of *C. cephalonica* were fully ground and mixed with the artificial diet for the brown planthopper. After the addition of *C. cephalonica* eggs, the preimaginal survival of *C. lividipennis* nymphs was significantly increased, from 55% to 81%. Thus, the quality of Diet-2 was significantly improved. Although nymphs fed on Diet-2 had a longer nutrient accumulation period than those fed on eggs of *N. lugens*, Diet-2 met the requirements of a Tier-1 assay. Before and after a 24 h exposure to *C. lividipennis*, the Cry1C protein in Diet-2 was stable and bioactive. This confirmed that Diet-2 could be used as a medium for detecting the dietary effects of Cry proteins on *C. lividipennis*.

Positive control compounds play important roles in dietary exposure assays. They can test whether insecticidal compounds are actually delivered into the gut of the test species, and they can determine whether the test system is able to detect treatment effects^16^,^17^. GNA is toxic to hemipterans and has potential applications in crop protection. Therefore, its action mechanism has received a great deal of attention^23^. In planthoppers, GNA binds to carbohydrate moieties on the cell surface, damages the microvilli brush border region of the midgut epithelium^24^, decreases the feeding, survival and fecundity of planthoppers, and retards planthopper development^25^. Like Cry toxins, GNA also binds to important midgut receptors of *N. lugens*, such as ferritin^26^. Based on these characteristics, GNA was used as a positive control compound in the present study and previous reports in Tier-1 tests^27^,^28^,^29^. Here, the survival of *C. lividipennis* fed Diet-2 containing increasing GNA concentrations significantly, but gradually, decreased compared with that of *C. lividipennis* fed the pure Diet-2 (*P* < 0.001). Similarly, the developmental duration was significantly prolonged by GNA, indicating that GNA is a proper positive compound for a Tier-1 assay of *C. lividipennis*. The Tier-1 system constructed in the current study was capable of detecting the dietary effects of insecticidal compounds. According to previous reports, there is a dose-dependent effect of GNA on *N. lugens*^30^. Ingestion of artificial diet containing 0.1% GNA (w:v) significantly decreased feeding and the honeydew excretion levels of BPH, however, after 24 h, there was some recovery in the honeydew excretion levels, and BPH appeared to tolerate the presence of GNA with time^31^. Whether the body-weights of *C. lividipennis* was not sensitive to GNA at a low dosage was caused by dose-dependent effects and tolerance of GNA needs to be further studied.

In the current study, 200 μg/ml Cry1C were added to Diet-2. This concentration was >10 times higher than the Cry content measured in transgenic *cry1C* rice (Table 6). This can be regarded as a worst-case exposure scenario, and it increased the possibility of detecting the potential detrimental effects of the Cry protein on *C. lividipennis*. Based on the results of the Tier-1 assays, in which more than 78% of *C. lividipennis* nymphs reached the adult stage when fed artificial diets containing the Cry1C protein, it is clear that *C. lividipennis* is not sensitive to Cry1C, and *C. lividipennis* could be expected to be not affected by the growing of Cry1C-expressing *Bt* rice. This Tier-1 system is more convenient and efficient than evaluating the biosafety of transgenic *Bt* rice through tritrophic prey-mediating assays, and it can be used to measure the effects of a broad-spectrum of Cry proteins on *C. lividipennis*.

When *N. lugens* nymphs or eggs and T1C-19 seedlings were provided simultaneously to *C. lividipennis*, Cry1C protein could, theoretically, be transferred to *C. lividipennis*. However, when the *N. lugens* nymphs or eggs reared with T1C-19 were provided to *C. lividipennis* without T1C-19, Cry1C was not detected in the predator. When *C. lividipennis* were maintained only with ‘T1C-19’ seedlings for one day, Cry1C was detected in *C. lividipennis*. This result is in accordance with the results of Han *et al*.^22^, showing again that Bt protein expressed by *Bt* rice could not be transmitted to *C. lividipennis* through predation on the eggs and nymphs of *N. lugens*, but instead was transferred by the piercing-sucking foraging behavior of *C. lividipennis* on rice. The valued biological functions and the special feeding behavior of *C. lividipennis* make it a good NTA surrogate for safety assessments of transgenic *Bt* rice.

High doses of Cry1C had no direct toxicity on *C. lividipennis*, and prey-mediated exposure to realistic doses of the Cry1C protein had no detrimental effects on the developmental time, preimaginal survival, female ratio or body weight of *C. lividipennis*. Additionally, the population density and population dynamics of *C. lividipennis* were not significantly affected by T1C-19. Thus, transgenic *cry1C* rice had no adverse effects on *C. lividipennis*. This is the first report of an assessment continuum for the effects of transgenic *cry1C* rice on *C. lividipennis*.

## Materials and Methods

### Rice materials

T1C-19 and Minghui 63 were used as rice materials for the experiments. T1C-19 is a transgenic *Bt* rice that expresses the *cry1C* gene under the control of the maize ubiquitin promoter. T1C-19 is highly resistant to lepidopteran insect pests on rice^14^. Minghui 63 is the non-transgenic isoline of T1C-19 that was used as the non-transgenic control. Both rice lines were provided by the National Key Laboratory of Crop Genetic Improvement, Wuhan, China. Yoshida culture solution^32^ was used for sustaining rice seedlings in the laboratory. Rice seedlings were cultured in plastic tanks (25-cm length × 620-cm width × 63-cm height), and 15-day-old rice seedlings (approximately 15 cm in height) were used for the experiment. The plants were grown at 26 ± 2 °C, relative humidity 80 ± 5% and light:dark cycle of 14 h:10 h.

### Insects

The original populations of *N. lugens, C. medinalis* and *C. lividipennis* were obtained from paddy fields in Wuhan, China. The independent colonies of *N. lugens* had been maintained on Minghui 63 and T1C-19 for more than 10 generations before the experiments. The eggs of *N. lugens* fed on Minghui 63 were used to rear *C. lividipennis*. More than six generations of *C. lividipennis* were continuously reared prior to the experiment. The *Bt*-susceptible strain of *C. medinalis* was maintained on maize for more than 10 generations. The *Bt*-susceptible strains of *Plodia interpunctella* (Hubner) (Lepidoptera: Pyralidae) and *C. cephalonica* were collected from the China Grain Reserves Corporation (Wuhan branch), and reared with artificial diets. The insects were maintained at 28 ± 1 °C, relative humidity 70 ± 5% and light:dark cycle of 14 h:10 h.

### Insecticidal compounds

The organic insecticidal compound GNA used in the present investigations was purchased from Sigma–Aldrich (St. Louis, Missouri, USA). The purity of GNA is ~90%. The molecular weight of GNA is 52 kDa. Lyophilized Cry1C protein was purchased from the Biochemistry Department Laboratory, School of Medicine Case Western Reserve University, USA. The purity of Cry1C is ~95–98% and the molecular size of the activated toxin is 65 kDa.

### Insecticidal bioactivity of Cry1C

Neonates of *P. interpunctella* were used to examine the bioactivity of this batch of Cry1C proteins. The toxicity of the Cry1C proteins to *P. interpunctella* was measured as described by Han *et al*.^22^. Cry1C was mixed with the artificial diet of *P. interpunctella* at concentrations of 0, 0.02, 0.05, 0.08, 0.11 and 0.14 μg/g, and supplied to *P. interpunctella* for seven days. Forty neonates for each repetition, with five repetitions, were used for each concentration. Based on the mortality of *P. interpunctella* larvae, the LC_50_ (concentration resulting in 50% *P. interpunctella* larval mortality as compared with the control) was measured. The LC_50_ of this batch of Cry1C was 0.03 μg/g fresh weight.

### Preparation of Diet-2 for *C. lividipennis*

Diet-2 for *C. lividipennis* larvae was developed based on the previously established artificial diet (Diet-1) used for rearing brown planthopper *N. lugens*^21^. Diet-2 for *C. lividipennis* was prepared according to the following procedure: (i) All Diet-1 ingredients, such as amino acids, vitamins, inorganic salts and sucrose, were prepared and completely dissolved; (ii) Eggs of *C. cephalonica* were fully ground using a mortar and pestle; (iii) The ingredients above were mixed and fully stirred (30 g ground eggs per 100 ml Diet-1); (iv) After centrifugation, the supernatant was collected; and (v) The solution was adjusted to pH = 6.8 with 4% of KOH and filter-sterilized through a Millipore disposable filter (0.45 μm). The diet was stored at −20 °C prior to its use in the experiments.

### Fitness of *C. lividipennis* feeding on Diet-2

**Figure 4:**
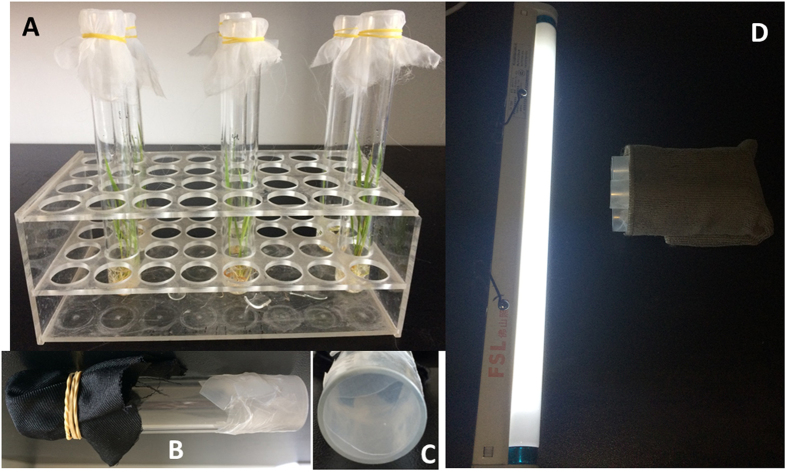
Rearing devices of *C. lividipennis* with natural food and artificial diet. (**A**) Rearing devices with natural food. 15-day-old rice seedlings were sustained with Yoshida culture solution, eggs or nymphs of *N. lugens* on rice seedlings were supplied as food of *C. lividipennis*; (**B**,**C** and **D**), rearing devices with artificial diet. Feeding chambers (**B**), artificial diet was held between two layers of stretched Paraffin film (**C**), and the feeding chamber was encircled with a wet dark brown towel except for the end containing Diet-2 being exposed to a light source (**D**).

To investigate whether Diet-2 could maintain the normal survival and development of *C. lividipennis*, a fitness bioassay was conducted in which *C. lividipennis* were fed either Diet-2 or a suitable natural food (eggs of *N. lugens*). For the natural food, two reproductive females were reared with 15-day-old Minghui 63 seedlings in a glass tube (3-cm diameter ×25-cm length). After laying eggs for two days, the *N. lugens* females were removed from the glass tube. Newly hatched *C. lividipennis* nymphs (<24 h) were reared individually with eggs of *N. lugens* in glass tubes covered with nylon mesh (Fig. 4A). From the first to the third instar stages of *C. lividipennis*, the preys were refreshed every two days. From the fourth instar to adulthood stages, the preys were refreshed every day. The survival and molting rates of the *C. lividipennis* nymphs were recorded every day. When the *C. lividipennis* adults emerged, the sex and body weights of these adults were recorded. In total, 32 individuals of *C. lividipennis* were tested.

For the artificial diet treatments, we used glass cylinders (12-cm length × 2-cm diameter) open at both ends as feeding chambers. Then, 100 μL of Diet-2 was held between two layers of stretched Paraffin film (stretched to about four times its original area) located at one open end of the feeding chamber, and the other open end was enclosed with wet black cloth (Fig. 4B,C). The first-instar nymphs of *C. lividipennis* were reared with *N. lugens* eggs as described above. When they molted into second instars (<24 h), the *C. lividipennis* nymphs were reared with Diet-2 individually in the feeding chamber. The feeding chamber was encircled with a wet dark brown towel except for the end containing Diet-2 being exposed to a light source (Fig. 4D). The diets were refreshed daily. The molting and survival of *C. lividipennis* were observed on a daily basis. Once adults emerged, they were sexed and weighed. Thirty-two nymphs of *C. lividipennis* were evaluated for each treatment.

### Validation of the dietary test system

GNA was incorporated into Diet-2 at concentrations of 0, 0.5, 1, 1.5 mg GNA/ml fresh diet, and *C. lividipennis* was reared with Diet-2 containing GNA. The selection of the GNA concentration range was based on a preliminary dose range-determining assay. The rearing procedure was the same as described above in the fitness bioassay. Thirty-two *C. lividipennis* nymphs were tested for each GNA concentration. The bioassays were terminated when all of the insects had developed to adults or had died in the control treatments.

### Stability and bioactivity of the Cry proteins in Diet-2

Prior to, and after 24 h, of feeding exposure for *C. lividipennis*, the Cry1C proteins in Diet-2 were extracted from the artificial diets, and AP005 ENVIRONLOGIX kits was used to determine the concentrations of Cry1C proteins remaining in Diet-2. We used this bioassay to examine the stability of Cry1C in Diet-2. Whether Cry1C is active during the feeding exposure duration also needs to be tested. Before and after 24 h of feeding exposure, Diet-2 containing 200 μg/ml Cry1C was diluted to 5 μg/ml and sprayed on the leaves of corn. After 2 h of air-drying, these treated corn leaves were supplied to *Bt*-susceptible second-instar larvae of *C. medinalis*, and the mortality rate of the insects was recorded 48 h later. Fifteen larvae for each replicate, with four replicates, were used for this bioassay.

### Effects of high doses of Cry1C protein on *C. lividipennis*

The dietary exposure assay was used to evaluate the direct toxicity of high doses of Cry proteins on *C. lividipennis*. Second-instar nymphs of *C. lividipennis* were individually fed with an artificial diet containing (i) Cry1C protein at 200 μg/ml of diet; (ii) GNA (positive control) at 1 mg/ml of diet; and (iii) no Cry1C (negative control). The molting and survival of *C. lividipennis* were observed daily. When the adults emerged, their genders and body weights were recorded. Thirty-two nymphs of *C. lividipennis* were evaluated for each treatment.

### Prey-mediated effects of transgenic *cry1C* rice on the life-table parameters of *C. lividipennis*

Eggs of *N. lugens* or newly hatched nymphs of *N. lugens* (24–48 h after hatching) fed on Minghui 63 or T1C-19 were supplied as prey to newly molted second-instar nymphs (<24 h) of *C. lividipennis*. The survival and molting of the *C. lividipennis* nymphs were monitored every day. After the adults of *C. lividipennis* emerged, the sex and body weights of these adults were recorded. The procedure was followed as described by Han *et al*.^22^.

### Cry1C contents in rice plants, *N. lugens* and *C. lividipennis*

Sheaths of T1C-19, eggs of *N. lugens* laid by adults fed on T1C-19 or Minghui 63, neonates of *N. lugens* fed on T1C-19 or Minghui 63 for two days, and third- or fourth-instar nymphs of *C. lividipennis* that preyed on eggs or nymphs of *N. lugens* fed on T1C-19 or Minghui 63 were collected as samples. The contents of Cry1C in the samples were determined using AP005 ENVIRONLOGIX kits (ENVIRONLOGIX, Portland, ME, USA). The determination protocol was as previously described^22^.

### The effects of T1C-19 on the *C. lividipennis* populations in rice fields

The population densities and population dynamics of *C. lividipennis* were investigated during the growing seasons of 2012–2013 in Hubei Province (Xiaogan City, 2012, 2013; Suizhou City, 2012). The field experimental design and sampling procedure were the same as described by Han *et al*.^22^. The population density of *C. lividipennis* was represented by the seasonal means as captured by vacuum-suction. The population dynamics of the predators were measured by the means at each sampling date.

### Data analysis

The significance of ELISA data, body weights, population densities and population dynamics between treatments were analyzed using Student’s *t*-tests. Preimaginal survival and female ratios were analyzed using Chi-square tests. Because the nymphal developmental time did not fulfill the assumptions required for parametric analyses (normal distribution of residues and homogeneity of error variances), it was analyzed using Mann–Whitney *U*-tests. Survival responses to the artificial diets containing insecticidal compounds were analyzed using the Kaplan–Meier procedure, and the log-rank test was used in the purified toxin experiment. The percentage data were arcsine–square-root transformed, and all count data were square-root (x + 1) or log_10_ (x + 1) transformed before being subjected to data analyses. The untransformed means are presented in the results. All statistical analyses were performed using the software package SPSS (version 16.0 for Windows, 2007).

## Additional Information

**How to cite this article:** Han, Y. *et al*. The tiered-evaluation of the effects of transgenic *cry1c* rice on *Cyrtorhinus lividipennis*, a main predator of *Nilaparvata lugens. Sci. Rep.* **7**, 42572; doi: 10.1038/srep42572 (2017).

**Publisher's note:** Springer Nature remains neutral with regard to jurisdictional claims in published maps and institutional affiliations.

## Figures and Tables

**Table 1:** Life-table parameters of *C. lividipennis* fed eggs of *N. lugens* or artificial diet.

Treatments	Percent nymphs	Developmental duration from	Adult fresh weight	
	developed to adults	2nd-instar to adults	(mg ± SE)^c^	
	(%)^a^	(days ± SE)^b^	Female	Male
Egg of *N. lugens*	90.6	7.8 ± 0.12 (29)	0.9 ± 0.04	0.5 ± 0.03
Diet-1	55.6	11.8 ± 0.40 (20)^**^	0.6 ± 0.02^**^	0.4 ± 0.02^**^
Diet-2	81.3	9.9 ± 0.19 (26)^**^	0.9 ± 0.03	0.5 ± 0.02

Note: (1) The experiment started with thirty-two 2^nd^-instar larvae per treatment, (2) Number of replicates is given in parentheses per treatment, (3) Asterisk denotes a significant difference between the natural food and artificial food: **<0.01. ^a^Chi-square test. ^b^Mann–Whitney *U*-test. ^c^Student’s *t*-test.

**Table 2:** Life-table parameters of *C. lividipennis* fed Diet-2 containing different concentrations of GNA.

Treatments	Percent nymphs	Development duration from	Adult fresh weight	
	developed to adults	2^nd^-instar to adults	(mg ± SE)^c^	
	(%)^a^	(days ± SE)^b^	Female	Female
Control (pure diet)	81.3	10.1 ± 0.13 (26)	0.80 ± 0.03 (14)	0.50 ± 0.02 (12)
GNA (0.5 mg/ml diet)	46.9^**^	11.4 ± 0.38 (15)^**^	0.80 ± 0.03 (8)	0.56 ± 0.03 (7)
GNA (1 mg/ml diet)	21.9^**^	11.6 ± 0.61 (7)^**^	—	—
GNA (1.5 mg/ml diet)	9.4^**^	14.3 ± 0.67 (3)^**^	—	—

Note: (1) The experiment started with thirty-two 2nd-instar nymphs per treatment, (2) Number of replicates is given in parentheses per treatment, (3) Asterisk denotes a significant difference between two diet treatments: ^**^P < 0.01. “–” indicates that number of replicates do not fulfill statistic analysis. ^a^Chi-square test. ^b^Mann–Whitney *U*-test. ^c^Student’s *t*-test.

**Table 3:** Effects of Cry1C and GNA in Diet-2 on life-table parameters of *C. livdipennis*.

Treatments	Percent nymphs	Developmental duration	Adult fresh weight	
	developed to adults	from 2^nd^-instar to adults	(mg ± SE)^c^	
	(%)^a^	(days ± SE)^b^	Female	Female
Control (pure diet)	84.4	10.2 ± 0.14 (27)	0.85 ± 0.03 (12)	0.57 ± 0.02 (15)
Cry1C (200 μg/ml diet)	78.1	10.3 ± 0.24 (25)	0.79 ± 0.03 (10)	0.55 ± 0.03 (14)
GNA (1 mg/ml diet)	25.0^**^	11.9 ± 0.40 (8)^**^	0.68 ± 0.04 (6)^*^	—

Note: (1) The experiment started with 322nd-instar larvae per treatment, (2) Number of replicates is given in parentheses per treatment, (3) Asterisk denotes a significant difference between diets containing insecticidal compounds and control: ^**^P < 0.01. ^a^Chi-square test. “–” indicates that number of replicates do not fulfill statistic analysis. ^b^Mann–Whitney *U*-test. ^c^Student’s *t*-test.

**Table 4:** Prey-mediated effects of Cry1C on life-table parameters of *C. lividipennis* preying eggs of *N. lugens* reared with *Bt* rice (T1C-19) or non-*Bt* (Minghui 63) rice plants.

Parameters	Rice line	
	T1C-19	Minghui 63
1st instar developmental time (days ± SE)^a)^	2.5 ± 0.06 (46)	2.5 ± 0.05 (46)
2nd instar developmental time (days ± SE)^a)^	1.7 ± 0.05 (43)	1.6 ± 0.04 (46)
3rd instar developmental time (days ± SE)^a)^	1.8 ± 0.06 (43)	1.9 ± 0.05 (43)
4th instar developmental time (days ± SE)^a)^	1.6 ± 0.07 (42)	1.6 ± 0.06 (42)
5th instar developmental time (days ± SE)^a)^	2.8 ± 0.08 (42)	2.7 ± 0.06 (41)
Whole larval stage developmental time (days ± SE)^a)^	10.4 ± 0.14 (42)	10.2 ± 0.10 (41)
Preimaginal survival (%)^b)^	91.3	89.1
Female ratio (%)^b)^	31	48.8
Male weight (mg ± SE)^c)^	0.53 ± 0.02	0.53 ± 0.03
Female weight (mg ± SE)^c)^	1.00 ± 0.04	0.99 ± 0.03

Note: (1) The experiment started with 46 nymphs per treatment. (n), number of individuals at each development stage. (2) Within a row, statistical comparisons were made for *Bt* rice with non-*Bt* rice. ^a)^Mann–Whitney *U*-test. ^b)^Chi-square test. ^c)^Student’s *t*-test.

**Table 5:** Prey-mediated effects of Cry1C on life-table parameters of *C. lividipennis* preying nymphs of *N. lugens* reared with *Bt* rice (T1C-19) or non-*Bt* (Minghui 63) rice plants.

Parameters	Rice line	
	T1C-19	Minghui 63
2nd instar developmental time (days ± SE)^a)^	1.9 ± 0.03 (50)	1.9 ± 0.03 (50)
3rd instar developmental time (days ± SE)^a)^	2.1 ± 0.06 (44)	2.0 ± 0.03 (44)
4th instar developmental time (days ± SE)^a)^	2.5 ± 0.07 (41)	2.4 ± 0.03 (41)
5th instar developmental time (days ± SE)^a)^	2.9 ± 0.12 (35)	2.8 ± 0.08 (38)
2nd instar-adult developmental time (days ± SE)^a)^	9.4 ± 0.19 (35)	9.1 ± 0.10 (38)
Preimaginal survival (%)^b)^	70	76
Female ratio (%)^b)^	45.7	42.1
Male weight (mg ± SE)^c)^	0.34 ± 0.05	0.31 ± 0.01
Female weight (mg ± SE)^c)^	0.48 ± 0.01	0.45 ± 0.05

Note: (1) The experiment started with 50 nymphs per treatment. (n), number of individuals at each development stage. (2) Within a row, statistical comparisons were made for *Bt* rice with non-*Bt* rice. ^a)^Mann–Whitney *U*-test. ^b)^Chi-square test. ^c)^Student’s *t*-test.

**Table 6:** Contents of Cry1C protein in rice sheath tissue, *N. lugens* and *C. lividipennis.*

Treatments	T1C-19	Minghui 63
Sheath of rice plants	1.8 ± 0.1 μg/g	Not detectable
Eggs of *N. lugens*	Not detectable	Not detectable
Nymphs of *N. lugens*	0.6 ± 0.05 ng/g	Not detectable
*C. lividipennis* provided with *N. lugens* eggs with rice plants	2.6 ± 0.3 ng/g	Not detectable
*C. lividipennis* provided with *N. lugens* nymphs with rice plants	0.6 ± 0.02 ng/g	Not detectable
*C. lividipennis* provided with *N. lugens* eggs without rice plants	Not detectable	Not detectable
*C. lividipennis* provided with *N. lugens* nymphs without rice plants	Not detectable	Not detectable
*C. lividipennis* provided with rice plants	3.9 ± 0.6 ng/g	Not detectable

Note: Data are represented as mean ± SE.

**Table 7:** Population densities (no. of per 0.25 m^2^) of *C. lividipennis* collected by vacuum-suction.

Rice materials	Xiaogan	
	2012	2013
T1C-19	1.29 ± 0.25	1.90 ± 0.58
Minghui 63	1.07 ± 0.16	1.29 ± 0.18

Note: N = 4 in 2012 and 2013. Data are represented as mean ± SE. There was no significant difference between *Bt* rice (T1C-19) and non-*Bt* rice (Minghui 63) field, based on Student’s *t-t*est.
